# Plaque biofilm microbial diversity in infants aged 12 months and their mothers with or without dental caries: a pilot study

**DOI:** 10.1186/s12903-018-0699-8

**Published:** 2018-12-29

**Authors:** Danying Tao, Fei Li, Xiping Feng, May Chun Mei Wong, Haixia Lu

**Affiliations:** 1 Department of Preventive Dentistry, Ninth People’s Hospital, Shanghai Jiao Tong University School of Medicine, National Clinical Research Centre of Stomatology, 500 Quxi Road, Shanghai, 200011 China; 2 Dental Public Health, Faculty of Dentistry, University of Hong Kong, 34 Hospital Road, Hong Kong, China

**Keywords:** Microbial diversity, Dental plaque, Sequencing analysis, Early childhood caries, Maternal influence

## Abstract

**Background:**

A number of studies on oral microbial diversity of early childhood caries (ECC) have tended to focus on mid- or late-stage of ECC, with a lack of research into early stage of tooth eruption and maternal influence. The aims of this study are to compare the supragingival plaque biofilm microbiota diversity between mothers with or without dental caries and their 12-month-old infants, and to explore the relationship of microbial diversity between infants and their mothers, using sequencing analysis.

**Methods:**

Supragingival plaque biofilm samples were collected from 20 pairs of mothers and their infants aged 12 months (10 mothers with dental caries and their 10 infants vs. 10 caries-free mothers and their 10 infants). The basic information of the mothers and infants had been collected through self-completed questionnaire. Pooled plaque biofilm DNA was extracted and DNA amplicons of the V4-V5 hypervariable region of the bacterial 16S rRNA gene were generated. Ilumina Miseq PE300 was used for 16S rRNA sequencing.

**Results:**

The results showed that high bacterial diversity was noted in the plaque biofilm of infants and their mothers with or without dental caries (dental caries mothers vs. caries-free mothers: 774 operational taxonomical units (OTUs) vs. 761 OTUs at a 3% divergence; infants whose mothers have dental caries vs. infants whose mothers are caries-free: 815 OTUs vs. 684 OTUs at 3% divergence). The Shannon microbial diversity index showed no statistically significant differences both on infants and their mothers between two groups (*p* > 0.05). Mother’s microbial diversity was higher than infants’ based on Shannon index (*p* < 0.05). Significant positive correlations were found between mothers’ and their infants’ Shannon index (*r* = 0.656, *p* = 0.002).

**Conclusion:**

Oral microbial diversity is significantly different between mothers and infants regardless of dental caries status, but no significant difference was found between mothers with and without dental caries or between their infants. Mother’s oral microbial diversity has an overall impact on the infants aged 12 months.

**Electronic supplementary material:**

The online version of this article (10.1186/s12903-018-0699-8) contains supplementary material, which is available to authorized users.

## Background

Early childhood caries (ECC) is one of the most common chronic diseases in preschool children, and it was about five times more common than asthma,which take the second place [1]. According to a systematic review published in 2016 [2], the prevalence of ECC at age 1 to 6 years in China was 0.3, 17.3, 40.2, 54.4, 66.1, and 70.7%, respectively, indicating that the prevalence of ECC gradually increases with age. Meanwhile, the prevalence of dental caries in very young children ranged from 28 to 82% [3–6]. Therefore, it is important to understand the natural history of ECC so that effective preventive strategies may be implemented. Early prevention and intervention of the initial stage of ECC is crucial as ECC adversely affects children physically and psychologically, and increases the economic burden on families and society [7].

Pathogenic microorganisms, not limited to *Streptococcus mutans,* has long been identified as an important and indispensable role in the development of dental caries [8]. However, the traditional culturing-dependent study of the cariogenic mechanism of microorganisms generally research pathogenic microorganisms separately. Since 1994, the ecological plaque hypothesis taking dental caries as a result of major disruption of the ecological balance in the oral microbial community is becoming increasingly accepted [9]. Under this theory, dental plaque behaves as a biological community and keeps a dynamic equilibrium with the host. Once the equilibrium is broken by the oral environment changes (such as with sugar intake, secretion of saliva, and changes of oral health status), bacteria within the biofilm may incline towards demineralization, resulting in demineralization even decay [10, 11].

Based on the ecological plaque hypothesis, more and more etiology researches on the cariogenic microorganism focused on ecological relationships within the biofilm, including microbial species diversity, genetic diversity, and functional diversity, rather than the certain types of microorganism. From this ecological perspective, oral cavity of the infant was found to be an excellent model to study the microbial diversity and dental caries etiology.

Oral cavity of newly born baby is generally sterile or contains only a few trespassing bacteria transmitted from vagina of their mother during childbirth [12]. Infant deciduous eruption creates a suitable environment for bacteria colonization, and after the eruption of teeth, the oral micro-ecological environment gradually becomes more complex, with numbers of microbes increasing and bacterial species increasing in complexity. Within one year of birth, species and numbers of oral microbial begin to increase with age, and get to stable until the eruption of permanent teeth. The bacteria colonization process from scratch provides a good blank control to verify the ecological plaque hypothesis.

Current etiology research of dental caries has transformed from a simple biomedical model into a broader, multidimensional and multifactor model, covering biological, environmental, behavioral, and social factors [13, 14]. More and more etiological studies of ECC have been carried out under the guidance of ecological plaque hypothesis. However, these studies always emphasize on the dental caries of infants and compare the microbial diversity between different level of dental caries in infants [15–21]. All along the child’s growth from birth to newborn feeding, the mother is the most frequent contacted person around the infant. Genetic methods have confirmed the vertical transmission of microorganisms from mother to infant [22]. Therefore, in any study of the cariogenic mechanism of microorganisms, as we should consider a variety of behavioral and social factors, the maternal influence cannot be ignored.

Guided by the ecological plaque hypothesis, the aim of the present study is to compare differences in the diversity of oral microorganisms between mothers with or without dental caries and their 12-month-old infants, using a new generation of high-throughput sequencing techniques. The overall impact of the oral microbial diversity of mothers with and without caries on the oral microbial diversity of their infants was also explored.

## Methods

### Study participants

The participants in the present study were 12-month-old infants and their mothers, which originally came from a previous study conducted by our research group. In the previous study, a number of pregnant women recruited from three maternal and child care service centers at a county level were invited to participant the study in Shanghai, China from October 2012 to March 2013. Their oral health-related quality of life during pregnancy was investigated and these specific contents have been described in detail elsewhere [23]. At one month and 12 months after giving birth, these mothers and their infants were followed up. A total of 20 pairs of 12-month-old infants and their mothers were selected based on inclusion and exclusion criteria, and assigned to an exposure group (mother with caries) or control group (mother without caries) according to the clinical findings on dental caries of their mothers when infants aged 12 months. There were 10 pairs in the exposure group and 10 pairs in the control group. Specific inclusion criteria for the exposure group were: (1) Mother participated in the previous project; (2) Mothers with one or more untreated dental caries when their infants aged 12 months; (3) Infant healthy, without hereditary diseases or deformities; (4) Existing tooth eruption in infant; (5) Infant did not take antibiotics within 2 months; (6) No history of dental treatment of infants.

An inclusion criteria of the control group were mothers without dental caries when the infants were 12 months old, and the remaining inclusion criteria of the exposure group (1 and 3–6) also applied to the control group.

The mothers participated in the study voluntarily and all signed written informed consent. For infants, parental written informed consent was also obtained. The study was conducted in accordance with the Declaration of Helsinki, and the protocol was approved by the Ethics Committee of the Ninth People’s Hospital, Shanghai Jiao Tong University prior to the implementation of the study (Project identification code: 201410).

### Questionnaire and clinical oral examination

When the infants reached 12 months, a follow-up questionnaire was self-completed by mothers, and clinical oral examinations of the mothers and their infants were carried out. Additionally, biofilm samples both from the mothers and infants were also collected for testing.

The basic information of the mothers had been collected through self-completed questionnaire during mother’s pregnancy and one month after the delivery of the infant. This information included socioeconomic status, mother’s own oral and general health related behaviors, and pregnancy outcomes. When the infant was 12 months old, questionnaire for the mother included infant’s feeding habits (such as breast or bottle feeding, use of nursing bottle at night, prolonged bottle feeding with sugar-containing drinks, sharing foods, drinks or utensils with infants), oral and general health-related behavior of mother as well.

Clinical oral examination for mother and their infant included dental caries level and oral hygiene status. Dental caries levels of mothers were diagnosed according to the criteria recommended by the WHO by a trained and licensed dentist [24]. Each examination was undertaken with a mouth-mirror and lightweight Community Periodontal Index (CPI) probe under artificial light. Dental caries detected visually at the cavitation level was recorded, and early caries was not recorded. All teeth present in the mouth (including third molars) were subjected to clinical oral examination. Dental caries of infants were divided into caries with cavity and early caries. Clinical manifestation of early caries is surface with opacities and discoloration when viewed as wet, while caries with cavity were assessed according to WHO recommendations [24]. The oral hygiene status of both mothers and infants were assessed using the visible plaque index (VPI) [25]. VPI was respectively scored as 0 or 1 corresponding to the absence or the presence of bacterial plaque on two surfaces per tooth; the percentage of the surfaces with plaque was also calculated.

### Biofilm acquisition and Total genomic DNA sample preparation

Biofilm sample were collected 2 h after eating or drinking both from mothers and infants. Supragingival plaques were sampled from all teeth presented in the month using a sterile excavating-spoon hand-instrument. For the mothers in the dental caries group, plaque from cavitated lesions was also collected. Pooled plaque samples were placed immediately in a sterile Eppendorf tube containing 1 ml sodium thiosulfate solution. These were then transferred to the laboratory with liquid nitrogen and stored in a ˗80 °C refrigerator.

Total genomic DNA extraction was performed using QIAamp DNA mini kit (Qiagen, Hilden, Germany) according to manufacturer’s instructions. Concentration and purity testing of the DNA was performed using a NanoDrop Minim 2000 spectrophotometer (NanoDrop Technologies, Wilmington, DE, USA).

### PCR amplification

Universal primers from 16S rRNA double V region (V4-V5) (515F 5’-GTGCCAGCMGCCGCGG-3′, 907R, 5’-CCGTCAATTCMTTTRAGTTT-3′) were used to for PCR amplification. After denaturation at 98 °C for 5 min, the main cycle, repeated 25 times, was carried out as follows: denaturation at 98 °C for 30 s, annealing at 50 °C for 30 s, extension at 72 °C for 30 s, final extension at 72 °C for 5 min. The quality of the amplified PCR products was detected by 2% agarose gel electrophoresis.

### Bioinformatics analysis

Ilumina Miseq PE300 sequencing platform (Ilumina, San Diego, CA, USA) was used for 16S rRNA sequencing of the amplified samples, with a depth of at least 30,000 sequences for each sample. Valid sequences were sorted and filtered from the original data. Quality double-ended FASTQ sequences were filtered by the sliding window method and Uchime method [26] in Mothur software (version 1.31.2, University of Michigan, USA) was used to obtain high-quality sequences.

Clustering of Operational Taxonomic Units (OTU) and subsequent bioinformatic analysis were performed at 3% divergence. High-quality sequences were clustered with QIIME software (Version 1.9.0, University of Colorado, Boulder USA), and the longest sequence was selected in each class as a representative sequence. RDP-classifier [27] and BLAST method in QIIME [28] were used to obtain taxonomic information for each OTU representative sequence.

Rarefaction curve and Good’s coverage were used to assess sequencing depth, and species accumulation curve was used to assess the adequacy of the sample size. Rank abundance distribution curve reflects the richness and evenness of species. The Shannon index [29, 30] is the most commonly used index to estimate microbe alpha diversity in the sample; a higher Shannon index value represents higher diversity of microbes.

The community composition of plaque samples is based primarily on the results of the OTU table, using QIIME software and database alignment to understand community structure in the samples at different taxonomic levels (phylum, class, order, family, and genus). Analysis of variance and multiple comparisons between the groups were performed to compare the dominant species on the genus level and to reflect differences in species richness. Principal component analysis (PCA) was used to assess beta diversity, classifying at the level of genus and species richness. R (vegan) software (version 3.1.2, University of Auckland, New Zealand) was used to draw a PCA diagram. The closer the distance between samples in the diagram, the more similar the microbial compositions in the samples were. One-way permutational multivariate analysis of variance (PERMANOVA) was calculated in QIIME software. Redundancy analysis (RDA) was performed using R (vegan) software.

Simultaneously, information on the 50 most-abundant genera was used to conduct correlation analysis between species, using the otu. Association command in Mothur. We applied R software for cluster analysis of classification information of the 50 most-abundant genera and created a heatmap chart.

## Results

### Basic information

A total of 20 pairs of 12-month-old infants and their mothers (10 pairs for exposure group and 10 pairs for control group) attended the present study. The mean age of mothers in the exposure and control groups was 28.5 ± 1.8 years and 27.1 ± 2.6 years, respectively (*p* = 0.182). There were no significant differences between groups in terms of mother’s sociodemographic background, mother’s oral health-related behaviors, infant birth histories, and infant feeding and oral health-related behaviors (all *p* > 0.05), indicating that the two groups were well matched. Mothers in the exposure group had at least one untreated dental caries, and the mean number of dental surfaces with caries (DS) was 9.2 ± 8.6. The number of dental surfaces with caries in mothers in the control group was 0, meaning that the difference in number of dental caries between the groups was statistically significant (*p* = 0.019). The VPI was not significantly different between the groups of mothers (0.5% ± 0.2% vs. 0.4% ± 0.2%, *p* = 0.184). All infants had anterior deciduous tooth eruption with no deciduous molar eruption observed and had no dental caries on sprouting tooth surfaces. The number of sprouting tooth surfaces, number of dental surfaces with caries, and VPI were not significantly different between the two infant groups (all *p* > 0.05).

A total of 40 supragingival pooled plaque biofilm samples were collected from the participants. All samples met quality inspection standards. Amplified PCR products were detected by 2% agarose gel electrophoresis and a representative gel image visualized under an ultraviolet light is shown in Additional file 1: Figure S1.

### Sequencing output

A total of 2,238,631 valid sequences were generated from a total of 40 DNA samples of supragingival plaque samples and 1,839,269 high-quality sequences remained for analysis after screening and optimization. From the sequencing samples (10 each) of the exposure and control mother groups, 480,598 and 432,829 high-quality sequences were obtained, respectively. From the samples (10 each) from the exposure and control infant groups, 512,131 and 413,711 high-quality sequences were obtained, respectively. The mean quantity of high-quality sequences from each sample was 45,982.

**Fig. 1:**
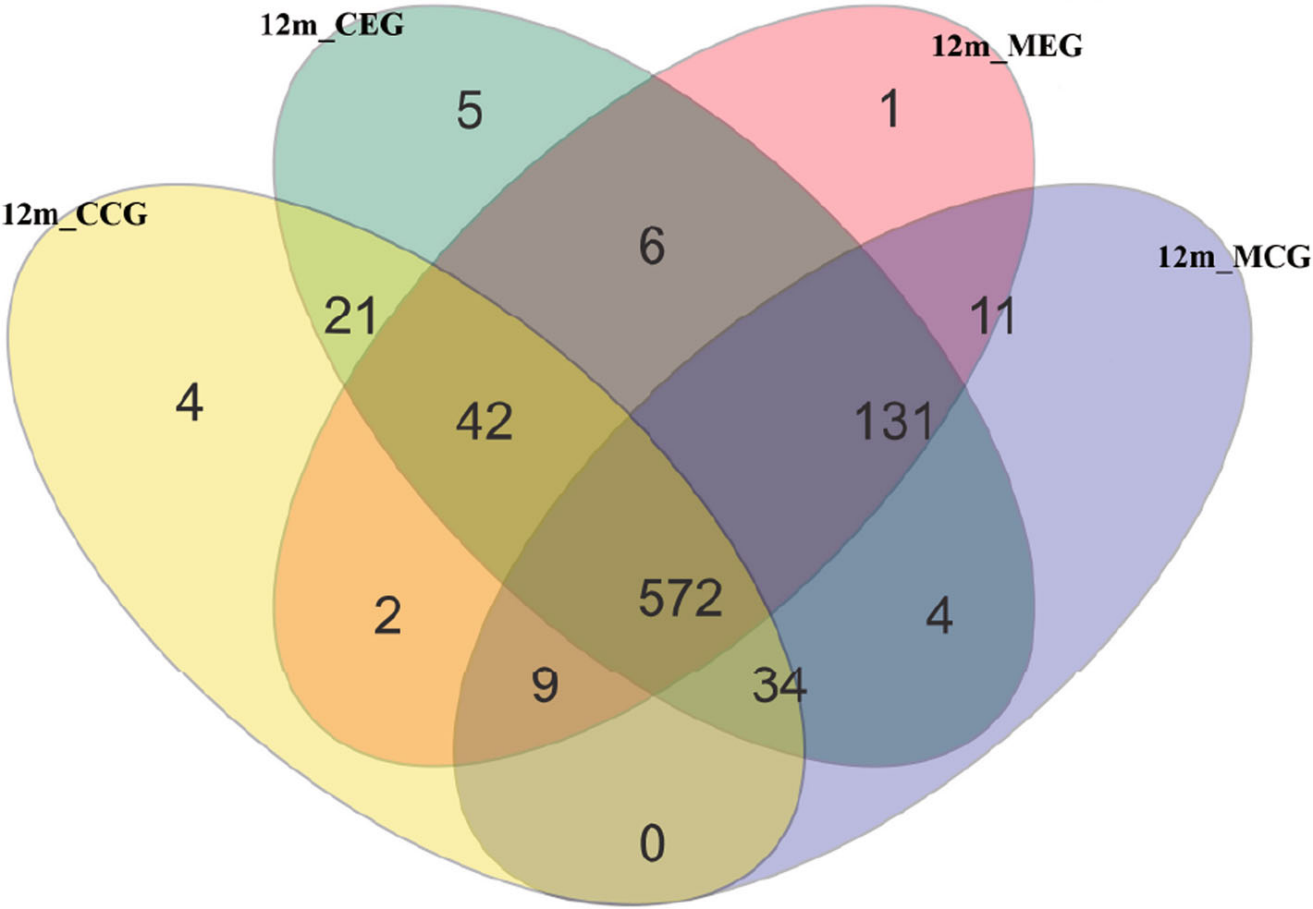
Venn diagram of OTU distributions among four groups. 12m_MEG: Mothers in the exposure group when children aged 12 months; 12m_CEG: Children in the exposure group when children aged 12 months; 12m_ MCG: Mothers in the control group when children aged 12 months; 12m_ CCG: Children in the control group when children aged 12 months

Sequence OTU clustering and notation were performed on the quality sequences at a 3% divergence level (Table 1). For the mothers in the exposure group (MEG), children in the exposure group (CEG), mothers in the control group (MCG) and children in the control group (CCG), OTU distributions are shown in a Venn diagram (Fig. 1). There were a total of 842 types of OTU in the four groups, with 572 OTUs shared among groups. OTU types shared by mothers across the two groups numbered 723, with 669 shared by the infants. A total of 751 OTU types were shared by mothers and infants in the exposure group, with 615 shared by mothers and infants in the control groups. OTU types of the four groups MEG, CEG, MCG and CCG, numbered 774, 815, 761, and 684, respectively.

**Table 1:** The results of OUT clustering

Sample	Groups	OTUs	Phylum	Class	Order	Family	Genus	Species
1011	MEG	339	339	337	333	321	270	77
1021	MEG	391	391	389	384	379	339	55
1031	MCG	372	372	370	362	355	303	74
1041	MEG	363	363	361	355	348	319	65
1051	MCG	364	364	363	357	351	311	64
1061	MEG	474	474	473	466	458	415	45
1071	MCG	430	430	428	422	414	356	65
1081	MEG	312	312	311	303	301	266	75
1091	MCG	391	391	389	382	370	323	77
1101	MCG	451	451	449	441	430	383	58
1111	MEG	343	343	342	333	325	281	75
1121	MEG	343	343	341	333	325	284	65
1131	MCG	363	363	361	354	344	298	68
1141	MEG	449	449	447	436	422	366	56
1151	MCG	365	365	364	355	346	292	71
1161	MEG	322	322	321	314	310	278	61
1171	MCG	398	398	396	389	380	331	60
1181	MEG	295	295	294	290	283	242	77
1191	MCG	395	395	393	381	367	301	78
1201	MCG	351	351	350	340	327	280	73
2011	CEG	357	357	356	352	338	277	71
2021	CEG	283	283	283	280	273	228	86
2031	CCG	254	254	253	250	242	195	92
2041	CEG	284	284	283	280	274	239	84
2051	CCG	180	180	180	177	174	144	100
2061	CEG	306	306	305	299	295	256	74
2071	CCG	298	298	297	291	284	234	78
2081	CEG	201	201	201	199	195	161	91
2091	CCG	316	316	314	311	305	269	69
2101	CCG	414	414	412	406	392	325	63
2111	CEG	298	298	297	293	286	241	78
2121	CEG	274	274	273	269	258	213	89
2131	CCG	279	279	278	273	266	228	84
2141	CEG	314	314	313	307	299	246	77
2151	CCG	193	193	192	188	183	159	87
2161	CEG	334	334	333	325	316	263	72
2171	CCG	273	273	273	270	258	216	87
2181	CEG	166	166	165	164	160	133	98
2191	CCG	284	284	282	277	272	235	83
2201	CCG	268	268	267	261	254	218	91

## Results based on species abundance

The rarefaction curve (Additional file 1: Figure S2) indicated that the sequencing depth of all the samples was reasonable and sequencing results reflected microbial information in the sample. The species accumulation curves (Additional file 1: Figure S3) already showed flattening when the number of samples was 10; with further increases in the number of samples, no more new OTUs can be obtained, indicating that the number of samples was reasonably set. The rank abundance curve for the samples (Additional file 1: Figure S4) is suggested that the diversity composition of oral microbiota represented in all samples had the characteristics of a small number of microorganisms being very abundant while the most microorganisms having low abundance.

**Fig. 2:**
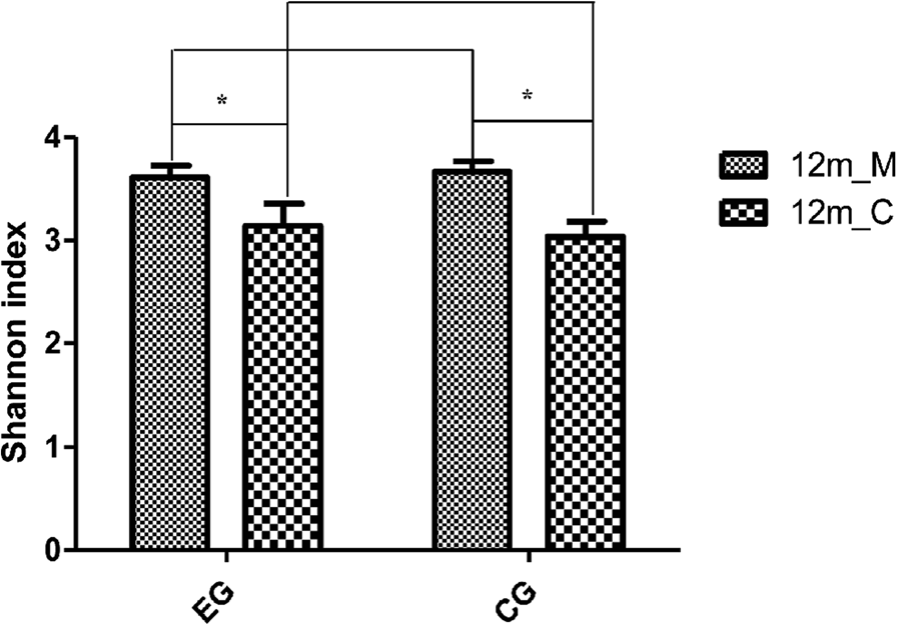
Comparison of Shannon index among four groups. 12m_M: Mothers when children aged 12 months; 12m_C: Children when children aged 12 months; EG: Exposure group; CG: Control group

The comparison of Shannon index for the four groups is shown in Fig. 2. The Shannon index of the mother groups was higher than that of the infant groups, indicating that the microbial diversity of the mother groups was higher than that of the infant groups. However, the Shannon index between the two mother groups was not significantly different (*p* > 0.05); the same result was observed between the two infant groups (*p* > 0.05). The Shannon indexes of mothers and infants in the exposure group were significantly different (*p* = 0.011); the same results was observed of mothers and infants in the control group (*p* = 0.020). Spearman correlation coefficient was used to assess the relationship of microbial diversity between mothers and their corresponding infants. Shannon indexes of mothers and infants were positively correlated (*r* = 0.656, *p* = 0.002). When oral microbial diversities of supragingival plaque biofilm of mothers was high, oral cavity diversity of the biofilm in their infants at the age of 12 months was also high; when the oral microbial diversity of the biofilm of mothers was low, that of their infants was also low.

## Results based on microbial community composition

**Fig. 3:**
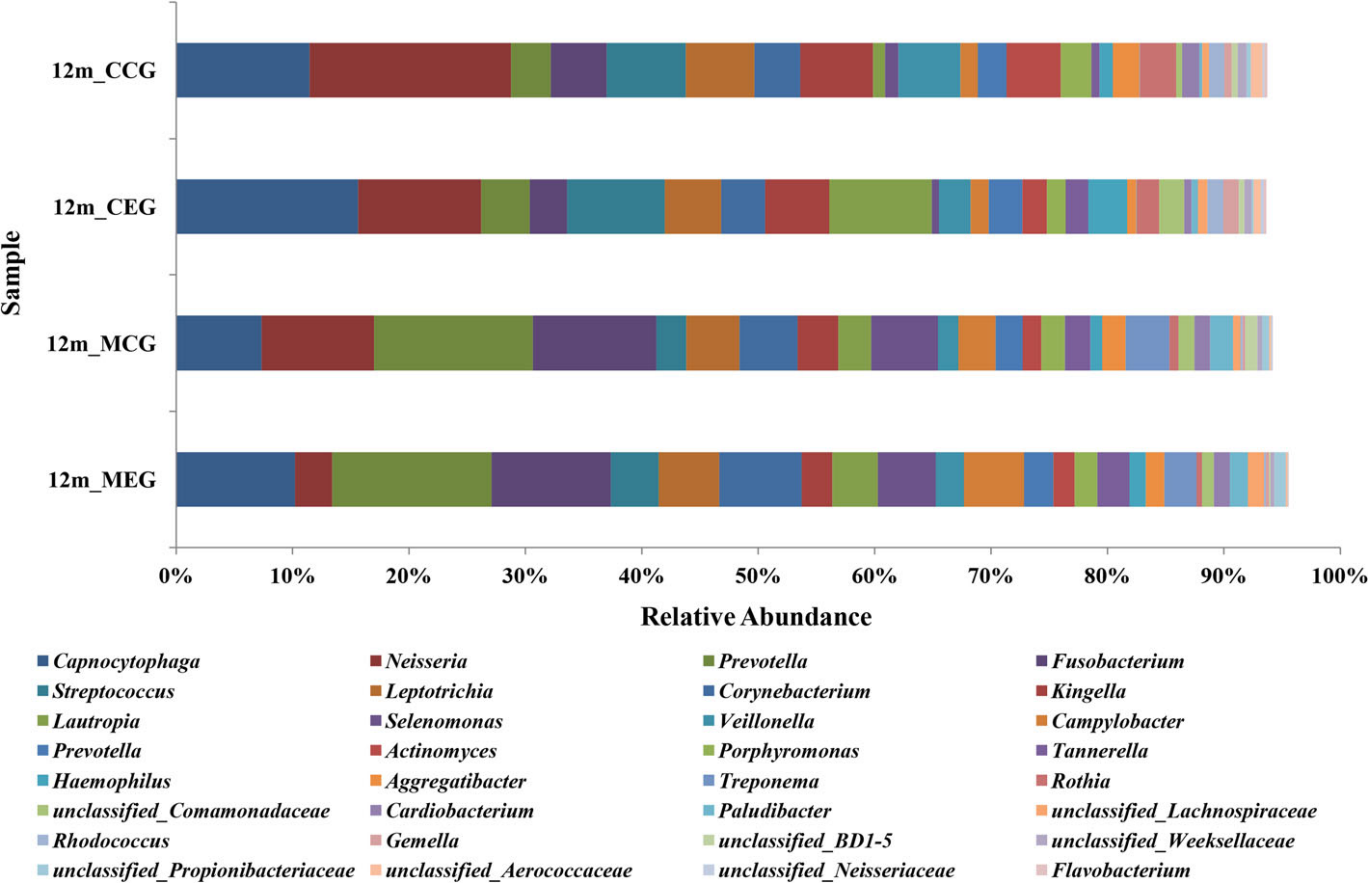
Classification of sample sequences at genus level

A total of 21 phyla, 36 classes, 57 orders, 94 families, 132 genera and 53 species were represented in four group plaque samples. The composition of four group samples with taxonomic classification at phylum level is shown in Fig. 3. A total of 21 microorganism phyla were found in four groups of samples: *Crenarchaeota, Euryarchaeota, Acidobacteria, Actinobacteria, Bacteroidetes, Chlamydiae, Chloroflexi, Cyanobacteria, Firmicutes, Fusobacteria, GN02, KSB3, Nitrospiraelanctomycetes, Planctomycetes, Proteobacteria, SR1, Spirochaetes, Synergistetes, TM7, Tenericutes,* and *Thermi*. Among them, six phyla were dominant in the oral microbial flora structure, accounting for more than 97% in each group; these were *Actinobacteria, Bacteroidetes, Firmicutes, Fusobacteria, Proteobacteria,* and *Spirochaetes*. In addition, *Nitrospirae, Chlamydiae,* and *Acidobacteria* were found only in the infants and not in the mother groups. *KSB3* was found only in the mothers group but not in either infant group.

At genus level of taxonomic classification, 198 different genera were found in the four sample groups. A total of 18 bacteria genera accounted for over 80% of the total microbiota (Fig. 3): *Capnocytophaga*, *Prevotella*, *Neisseria*, *Fusobacterium*, *Corynebacterium*, *Leptotrichia*, *Streptococcus*, *Kingella*, *Selenomonas*, *Lautropia*, *Campylobacter*, *Veillonella*, *Actinomyces*, *Porphyromonas*, *Treponema*, *Tannerella*, *Paraprevotellaceae*, and *Rothia*. In all the 4 groups, the distribution of the dominant species was similar, However, the proportion of core genera showed difference. *Neisseria*, *Streptococcus and Kingella* were more abundant in the infants groups, and *Prevotella, Fusobacterium, Selenomonas* showed advantage in the mother groups.

Results of Analysis of Variance for the abundance level of the 18 dominant genera are shown in Table 2. The significant abundance difference of dominant bacteria was not showed between mothers with caries and without caries. The dominant genus that differed in abundance between the two infant groups was *Lautropia*. The dominant genera with differing abundance between mothers and infants in the exposure groups included *Capnocytophaga*, *Prevotella*, *Fusobacterium*, *Corynebacterium*, *Streptococcus*, *Kingella*, *Selenomonas*, *Campylobacter*, and *Treponema*. The dominant genera with a difference in abundance between mothers and infants in the control groups include *Prevotella*, *Selenomonas* and *Treponema*.

**Table 2:** Differences of relative abundance of predominant bacteria by genus

Predominant genus	MEG & MCG	CEG & CCG	MEG & CEG	MCG & CCG
*Capnocytophaga*	0.514	0.057	0.027*	0.326
*Prevotella*	0.688	0.58	0.024*	0.002*
*Neisseria*	0.190	0.422	0.083	0.216
*Fusobacterium*	0.992	0.639	0.032*	0.086
*Corynebacterium*	0.522	0.623	0.032*	0.375
*Leptotrichi*	0.876	0.642	0.853	0.663
*Streptococcus*	0.605	0.131	0.023*	0.188
*Kingella*	0.535	0.447	0.009*	0.176
*Selenomonas*	0.436	0.703	0.002*	0.001*
*Lautropia*	0.975	0.014*	0.078	0.435
*Campylobacter*	0.090	0.729	0.005*	0.113
*Veillonella*	0.964	0.568	0.595	0.254
*Actinomyces*	0.993	0.305	0.489	0.092
*Porphyromonas*	0.865	0.715	0.795	0.783
*Treponema*	0.485	0.998	0.012*	0.002*
*Paraprevotellacea*	0.794	0.481	0.738	0.912
*Rothia*	0.656	0.584	0.108	0.089

*p value <0.05, showed that significant abundance difference of dominant bacteria was found among four groups

*MEG* Mothers in the exposure group, *CEG* Children in the exposure group, *MCG* Mothers in the control group, *CCG* Children in the control group

**Fig. 4:**
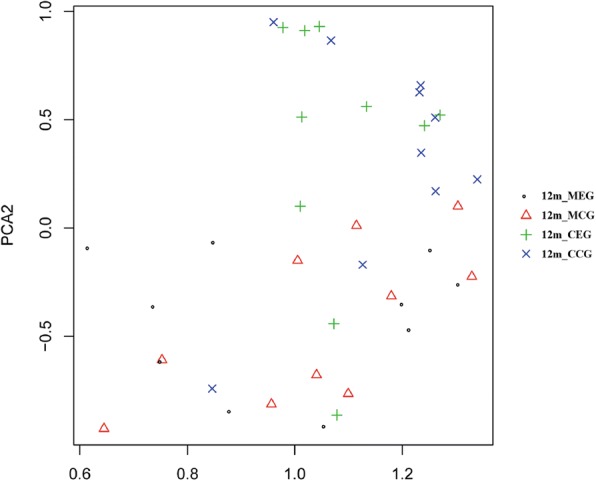
PCA analysis

PCA of 40 samples in four groups is shown in Fig. 4. The microbiome profiles of dental plaque samples did not show significant differences between the two mother groups (F = 1.2, *p* > 0.05) or the two infant groups (F = 1.2, *p* > 0.05). However, significant differences was observed between the samples derived from the infants and those from mothers (F = 5.8, *p* = 0.001 for exposure group and F = 1.6, *p* = 0.002 for control group), indicating that the community microbial structures of 12-month-old infants and mothers differ on the overall.

**Fig. 5:**
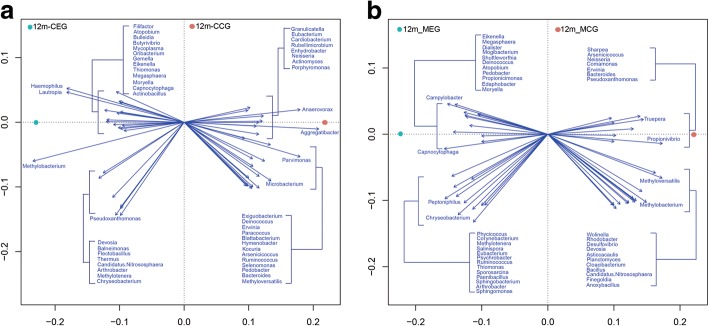
RDA analysis

To identify the general-level microbiota phylotypes associated with the different dental caries status of mothers, redundancy analysis (RDA) was performed for OTUs from two groups of infants and two groups of mothers (Fig. 5). The dental caries status of mothers was used as environmental variables and the relative abundances of all OTUs in the plaque samples were used as species variables. Monte Carlo Permutation Procedure (MCPP) showed that the microbiota differences from the two infants groups (*p* = 0.868) and two mothers groups (*p* = 0.797) were both insignificant. 50 generals explaining more than 4% of the variability of the microbiota as key phylotypes associated with dental caries was identified respectively in the two infants groups and two mothers groups.

**Fig. 6:**
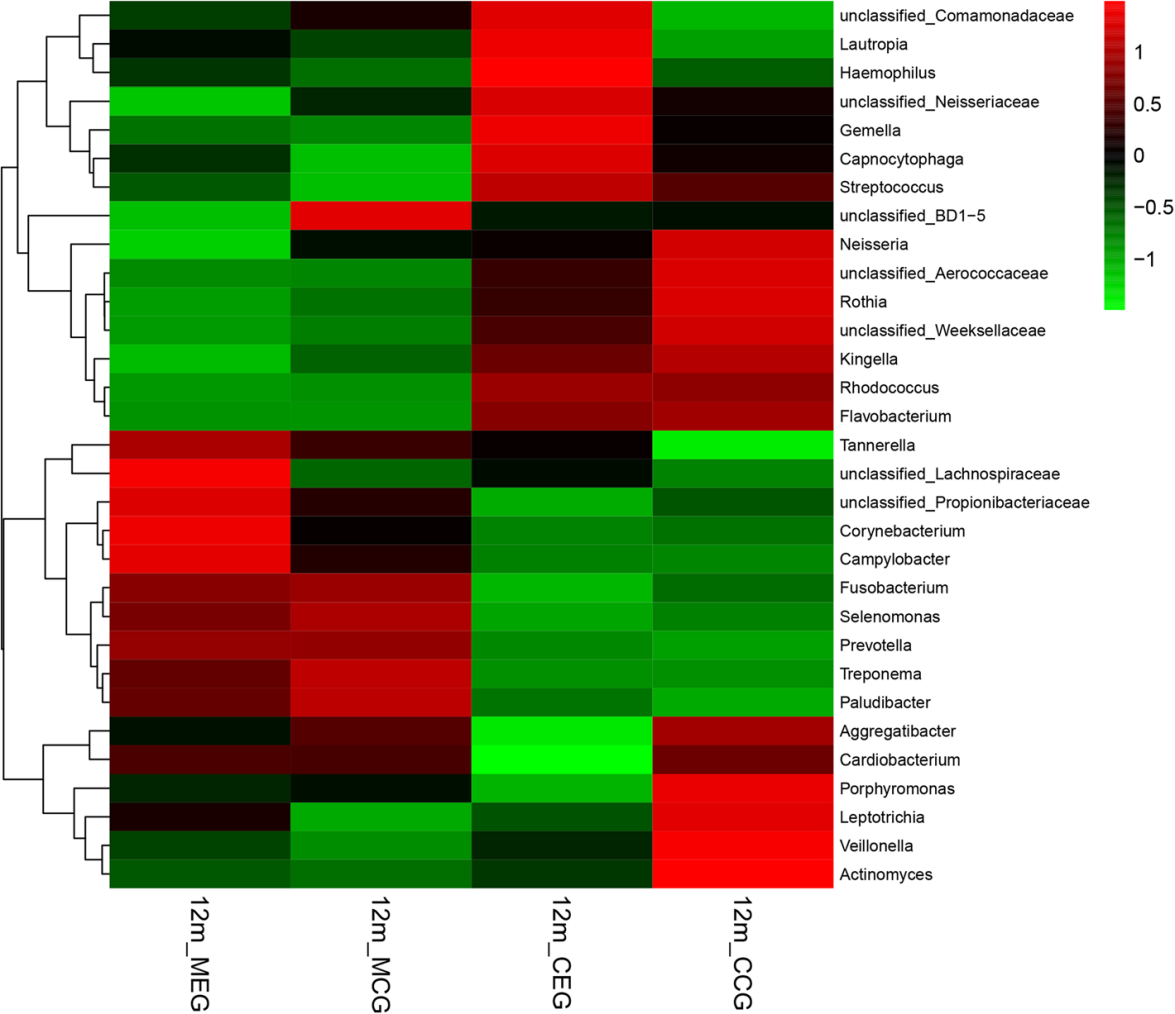
The heatmap chart and clustering results of 50 most-abundant bacterial genera

The heatmap of the 50 most-abundant genera in the four sample groups is shown in Fig. 6. Clustering results showed that the overall distribution of the dominant species could be easily told apart between the mother group and the infants group, while the variation within group was not significant. Samples derived from infants are relatively close in the cluster, and the same is true for the majority of samples from the mothers.

## Discussion

Although the current prevalence of ECC is high, the distribution of deciduous caries is by no means even, with high- and low-risk groups. Therefore, early screening of child population at risk of dental caries is particularly important for primary prevention. Despite a number of previous studies on the plaque biofilm of ECC, there is a lack of overall, comprehensive and dialectical understanding of the relationship between dental caries and oral microbes from an ecological perspective. With the rise of an ecological plaque hypothesis for dental caries, study of the composition and characteristics of microbial biofilm from an overall perspective has become the focus. Previous studies of ECC ecology have tended to focus on mid- or late-stage ECC [15–17, 20, 31, 32], with a lack of research into early stage of tooth eruption. To fill these research gaps, the present study, under the guidance of an ecological plaque hypothesis, compared oral microbial diversity related to dental caries in 12-month-old infants and their mothers, and explored the overall effect of the oral microbial diversity of mothers with and without caries on infant oral microbial diversity.

At a 3% divergence level, OTU types from the four groups, MEG, CEG, MCG, CCG numbered 774, 815, 761, and 684, respectively. The highest number of OTU types came from samples of infants in the exposure group. Cephas et al. [33] used 454 pyrosequencing and found that the detection rate of oral streptococci in edentulous children was significantly higher than that in adults. Although the number of oral microorganisms detected in infants was less, their oral microbial diversity was higher than that of their parents. The findings in the present study, that infants in the exposure group have the highest number of OTUs types, is similar to Cephas et al.’s study. The infant immune system is immature and the plaque ecosystem is still in a process of gradual evolution and maturity. Trespassing bacteria present in the short term in the mouth may be the reason for the presence of greater numbers of oral microbial species in infants than in adults. In the present study, a set of micro-organism phyla (*Nitrospirae*, *Chlamydiae*, and *Acidobacteria*) were found only in the infants and not in the mother groups. A relatively low proportion of these two phyla (*Nitrospirae* and *Acidobacteria*) has also been found in the children aged 3–6 years regardless of their dental caries status in the Jiang’s study [19], indicating that these two phyla may be the composition of microorganism in oral cavity even with increasing age. However, as to the *Chlamydiae*, no other ECC ecological studies found this phylum. The possible explanation is that *Chlamydiae* are a transient microorganism, and then gradually disappear or to be very low proportion in oral cavity with the eruption of posterior teeth.

Shannon index was calculated for each group to compare the oral microbial diversity in each group. PCA analysis was also used to determine the overall similarity between microbial communities in each sample group. RDA analysis was performed to identify the species-level microbiota phylotypes associated with the different dental caries status of mothers and infants. Comparison of Shannon index gave no significant difference between the two groups of mothers and infants. PCA analysis showed that microbial community compositions in supragingival plaque biofilm were similar between the mothers in the exposure and control groups. The same was true of infants in the exposure and control groups. Results of the RDA analysis also showed that no microbiota phylotypes differences were found between the infants from two groups and mothers from two groups. PCA analysis also showed that community compositions of most samples from the infants differed from the mothers, with comparison of Shannon index revealing significant differences between the two groups of mothers and their infants. These results indicated PCA analysis and Shannon index results were comparable, stable and consistent for the assessment of microbial diversity.

With matched social and behavioral aspects in relation to dental caries, it was found that oral microbial diversity between mothers and their 12-month-old infants was positively correlated, suggesting that the oral microbial communities of mothers has an effect on the oral microbial communities of infants. Caufield et al. [34] and Alves et al. [22] have been confirmed that the vertical transmission of microorganisms between mother and infant is existed. Previous studies indicated a strong association between *S. mutans* acquisition of infants and maternal *S. mutans* levels. Similar or identical bacteriocin profiles were found between infants and mothers [35]. Infants can be colonized with cariogenic bacteria (mainly on *S. mutans*) during the pre-dentate stage, with some infants colonized as early as 3 months of age [35, 36]. However, previous research has been limited mostly to investigate on the vertical transmission of specific types of bacteria such as *S. mutans*. To the best of our knowledge, this is the first study on the microbial diversity relationship between mothers and their infants exploring the overall microflora. The results also suggest that the mother’s oral microorganisms should not be ignored when screening high-risk populations of young children for dental caries.

Some feeding habits, especially on use of nursing bottle at night, prolonged bottle feeding with sugar-containing drinks, as well as frequency use of snacking are well-documented factors which are associated with bacterial level and development of dental caries in young children [37]. Mother who share food, drinks or utensils with their infants have the higher risk of transmitting *S. mutans to* their children [38, 39]. Feeding practices including breast feeding or bottle feeding were also considered to be risk factors of ECC. However, contradictory finding that no significant effect of either breast-feeding or bottle-feeding on ECC were also found [40]. In the present study, information on infants’ feeding habits such as breast or bottle feeding, use of nursing bottle at night, prolonged bottle feeding with sugar-containing drinks as well as share food, drinks or utensils with their infants was collected through questionnaire. The results showed that there were no significant differences between groups in terms of these feeding habits, indicating that the two groups were well matched. Therefore, the influence of feeding habits on the microbial diversity of infants would consider to be ignored.

Throughout childhood, oral microorganisms constantly change, evolve, and mature. Our abundance analysis showed that the dominant genus with abundance differences between two infant groups was only one, *Lautropia*, while Shannon index scoring and the RDA analysis result also revealed that oral microbial diversity was not significantly different between the two infant groups. This suggests that, at the same age, the plaque biofilms of infants have a similar degree of evolution and maturity.

In the past, high-throughput sequencing studies on ECC comparing microbial diversity at caries and caries-free sites have resulted in the proposal of a number of microorganisms related to ECC. Ling et al. [20] indicated that *Streptococcus*, *Veillonella*, *Actinomyces*, *Granulicatella*, *Leptotrichia*, and *Thiomonas* were all related with ECC. Kanasi et al. [15] suggested that 139 types of different microorganisms were associated with ECC. Jiang et al. [19] believed that three genera, *Streptococcus*, *Granulicatella*, and *Actinomyces,* were related to ECC. Previous studies on caries microorganisms using sequencing technology tended to conclude that some microorganisms may be related to the occurrence of dental caries [41], or that microbial diversity at caries sites is less than that at healthy sites [19]. The present study did not find the dental caries in infants, and *Neisseria*, *Streptococcus* and *Kingella* were more abundant genera in the infants groups. This finding was in accordance with the results of study among 3-years-old caries-free children, in which the high frequency genera were *Kingella*, *Capnocytophaga*, and *Neisseria* [42]. Recently, a longitudinal study was conducted to monitor the dynamic changes in the structural composition of oral microorganisms of a cohort of 3-year-old children during the process of caries development [42]. The results showed that two major shifting patterns in microbial structures during caries development in caries-affected group, whereas no obvious shifting patterns were observed in caries-free group. These would enhance our understanding of ECC-related microbes and the comprehensiveness of the ecological plaque hypothesis.

A RNA-based microbial study demonstrated that the composition of active bacteria in enamel lesions appears to be different from that found in more advanced dentine cavities, suggesting that these microbial communities are tissue dependent [43, 44]. The dentine cavities of a molar tooth had more abundance of *Lactobacillus*, *Shlegelella*, *Pseudoramibacter*, and *Atopobium*, whereas an initial enamel caries lesion had high levels of *Streptococci*, *Rothia*, *Leptotrichia*, and *Veillonella.* The same research group also conducted a metagenomic study indicated that enamel caries bacteria have an over-representation of dietary sugar-fermenting genes, while dentin caries organisms are enriched in genes participated in the metabolism of human-associated glycans [45]. Furthermore, enamel caries microorganisms are extremely rich in adhesion molecules whereas the microbial community in dentin caries contains a remarkable arsenal of proteases to degrade dentinary tissue, including collagenases, dipeptidyl peptidases, serine proteases, glycoproteases, matrix metallopeptidases, and aminopeptidases. In the present study, *Prevotella*, *Fusobacterium*, *Selenomonas* showed advantage in the mother groups and the abundance differences of dominant genera showed no differences between mothers with caries and those without. Also the Shannon index and RDA analysis results of oral microbiota between the two groups also showed no difference. The difference in results may be due to little discrepancy of the mean number of dental surfaces with caries between two mothers groups. Although the mean number of dental surfaces with caries between mothers with or without dental caries was 9.2 Vs. 0 (*p* = 0.019), the discrepancy on microbial diversity may be not large enough to distinguish. More comprehensive study design, say three groups for mother (caries free group, low caries group and high caries group) may be adopted in the future study.

There are some other limitations needed to be addressed. First of all, the present study included the 20 pairs of mothers and their infants aged 12 months, with 10 mothers with dental caries and their 10 infants vs. 10 caries-free mothers and their 10 infants. Although the species accumulation curves showed that no more new OTUs can be obtained with further increases in the number of sample size, indicating that the number of samples was reasonably set, it would be better to increase the sample size to discriminate the difference between two groups. Secondly, the observation interval is the infants aged 12 months, and no dental caries was found for all infants when they were 12 months of age, which may lead to difficult to observe the endpoint of microflora succession and analyze early cariogenic bacteria. In the future, it is necessary to further prolong the observation period.

## Conclusions

Oral microbial diversity is significantly different between mothers and infants regardless of dental caries, but no significant difference was found between mothers with and without caries or between their infants. Oral microbial diversity between mothers and their 12-month-old infants showed a significant positive correlation, indicating that the mother’s oral microbial diversity has an overall impact on that of her child, which further confirmed the vertical transmission of cariogenic microorganisms between mother and infant from the ecological perspective.

## Additional file

Additional file 1: Supplementary figures for the manuscript. **Figure S1.** An example of a gel electrophoresis diagram of PCR products. **Figure S2.** Rarefaction curves of samples. **Figure S3.** Species accumulation curves. **Figure S4.** Rank abundance curve. (DOC 388 kb)

## Abbreviations

CCG: Children in the control group
CEG: Children in the exposure group
CPI: Community Periodontal Index
ECC: Early childhood caries
MCG: Mothers in the control group
MCPP: Monte Carlo Permutation Procedure
MEG: Mothers in the exposure group
OTUs: Operational taxonomical units
PCA: Principal component analysis
RDA: Redundancy analysis
VPI: Visible plaque index
