# Supplementary material for: Plaque biofilm microbial diversity in infants aged 12 months and their mothers with or without dental caries: a pilot study
Source: BMC Oral Health. 2018 Dec 29;18:228. doi: 10.1186/s12903-018-0699-8 (PMC6311051; doi:10.1186/s12903-018-0699-8)
Supplement: Supplementary file 1 — Supplementary figures for the manuscript. Figure S1. An example of a gel electrophoresis diagram of PCR products. Figure S2. Rarefaction curves of samples. Figure S3. Species accumulation curves. Figure S4. Rank abundance curve. (DOC 388 kb) [file 12903_2018_699_MOESM1_ESM.doc]

**Plaque biofilm microbial diversity in infants aged 12 months and their mothers with or without dental caries: a pilot study**

Danying Tao^1^, Fei Li^1^, Xiping Feng^1^, May. Chun. Mei. Wong^2^, Haixia Lu^1^*

^1^ Department of Preventive Dentistry, Ninth People’s Hospital, Shanghai Jiao Tong University School of Medicine, Shanghai Key Laboratory of Stomatology & Shanghai Research Institute of Stomatology; National Clinical Research Centre of Stomatology, 500 Quxi Road, Shanghai, China

^2^ Dental Public Health, Faculty of Dentistry, University of Hong Kong, 34 Hospital Road, Hong Kong, China

[taodanying@sina.com](mailto:taodanying@sina.com) (D. T.); [charlse123@163.com](mailto:charlse123@163.com) (F. L.); [fengxiping9h@163.com](mailto:fengxiping9h@163.com) (X. F.); [mcmwong@hku.hk](mailto:mcmwong@hku.hk) (M. C. M. W.); [Ritalu0225@hotmail.com](mailto:Ritalu0225@hotmail.com) (H. X.).

*Correspondence:

Dr. Haixia Lu,

Address: Department of Preventive Dentistry, Ninth People’s Hospital, Shanghai Jiao Tong University School of Medicine, 500 Quxi Road, 200011, Shanghai, China.

E-mail address: ritalu0225@hotmail.com

**Additional file 1: Supplementary figures for the manuscript.**

Figure S1. An example of a gel electrophoresis diagram of PCR products.

Figure S2. Rarefaction curves of samples.

Figure S3. Species accumulation curves.

Figure S4. Rank abundance curve.

**
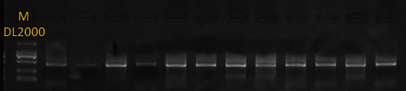
**

**Figure S1**

**
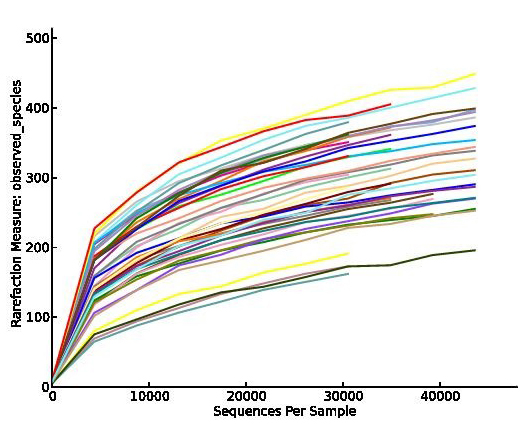
**

**Figure S2**

**
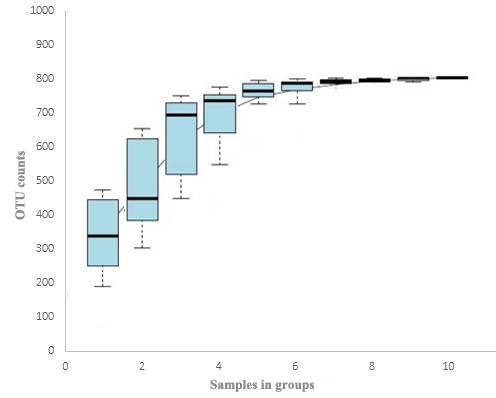
**

**Figure S3**

**
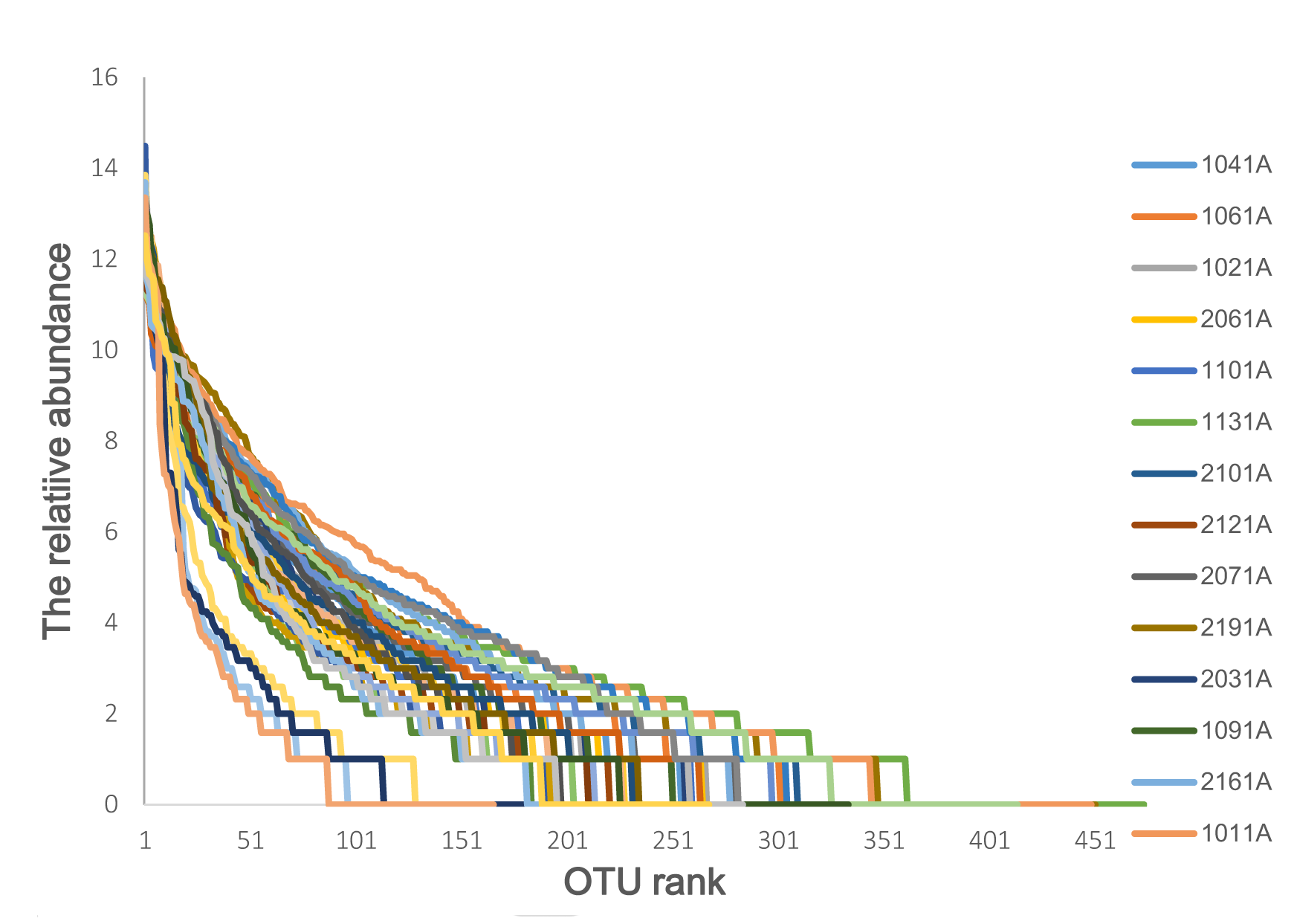
**

**Figure S4**
